# High-throughput, nonperturbing quantification of lipid droplets with digital holographic microscopy

**DOI:** 10.1194/jlr.D085217

**Published:** 2018-04-05

**Authors:** Vasco Campos, Benjamin Rappaz, Fabien Kuttler, Gerardo Turcatti, Olaia Naveiras

**Affiliations:** Laboratory of Regenerative Hematopoiesis (GR-NAVEIRAS)* École Polytechnique Fédérale de Lausanne (EPFL), Lausanne, Switzerland; Biomolecular Screening Facility,† École Polytechnique Fédérale de Lausanne (EPFL), Lausanne, Switzerland; Hematology Service, Department of Oncology,§ Centre Hospitalier Universitaire Vaudois (CHUV), Lausanne, Switzerland

**Keywords:** adipogenesis, adipocyte, label-free

## Abstract

In vitro differentiating adipocytes are sensitive to liquid manipulations and have the tendency to float. Assessing adipocyte differentiation using current microscopy techniques involves cell staining and washing, while using flow cytometry involves cell retrieval in suspension. These methods induce biases, are difficult to reproduce, and involve tedious optimizations. In this study, we present digital holographic microscopy (DHM) as a label-free, nonperturbing means to quantify lipid droplets in differentiating adipocytes in a robust medium- to high-throughput manner. Taking advantage of the high refractive index of lipid droplets, DHM can assess the production of intracellular lipid droplets by differences in phase shift in a quantitative manner. Adipocytic differentiation, combined with other morphological features including cell confluence and cell death, was tracked over 6 days in live OP9 mesenchymal stromal cells. We compared DHM with other currently available methods of lipid droplet quantification and demonstrated its robustness with modulators of adipocytic differentiation in a dose-responsive manner. This study suggests DHM as a novel marker-free nonperturbing method to study lipid droplet accumulation and may be envisioned for drug screens and mechanistic studies on adipocytic differentiation.

Considering the prevalence of obesity and its rising burdens on the health and costs of Western societies (1, 2), understanding the mechanisms underlying adipocyte physiopathology has become increasingly important. Central to the nature of adipocytes is the differentiation and subsequent lipid droplet accumulation in mesenchymal stromal cells (MSCs) and preadipocytes (3–5). Novel methods that are robust enough to detect early stages of differentiation would accelerate our understanding of metabolic diseases and other disorders involving lipid accumulation.

Various methods have been used to quantify adipogenesis in vitro (6–12). However, they all rely on liquid handling steps, including fixation, diverse staining, imaging, and image analysis procedures, which all require lengthy process optimizations and are therefore difficult to reproduce quantitatively (13). Other methods rely on RNA quantification or biochemical assays, but these fail to capture the morphological changes and single cell properties that define adipogenesis. One of the main challenges when assessing adipocytic differentiation is the extreme fragility of mature adipocytes. Not only are adipocytes sensitive to temperature fluctuations or oxygenation, but they also have a natural tendency to detach and float, which increases variation and quantification biases (14). In order to avoid procedural biases, it has become standard to detect and assess adipocyte differentiation at early stages by using rapidly differentiating clonal lines (6). This approach limits our understanding of the mechanisms of adipocyte differentiation to the existing lines and may fail replication in alternative lines. In addition, improving adipocyte differentiation detection methods would be compatible with measurements at earlier time points. Here, we report a new method that enables detection of lipid droplets in differentiating adipocytes, without the need for washing, staining, or other liquid manipulations. Digital holographic microscopy (DHM) allows for the reconstruction of images based on the ability of tissue to refract light (15, 16). While the sample is illuminated with a laser emitting a specific wave, a reference beam passes in parallel to the sample, and the combination of both waves creates a hologram encoding phase shift information (17). Quantitative phase images are then numerically reconstructed from the hologram, which contains morphological and intracellular information related to the refractive index of the monitored cells. Lipid droplets have a higher refractive index, producing a very strong contrast, and are thus easily quantified. The ability to numerically “refocus” the image postacquisition allows for rapid and noninvasive sampling (18). In this study, we used the bone marrow-derived MSC line OP9 (19), as it is both described as a model of rapid adipocytic differentiation and it was derived nonclonally, allowing us to assess the heterogeneity of cells undergoing adipocytic differentiation (20, 21). We compared the ability of DHM to capture adipocytic differentiation with other existing quantification methods, including flow cytometry (22) and fluorescence imaging. We performed a time-lapse analysis of OP9 adipocytic differentiation and tested the capacity of DHM to capture modulations of adipocytic differentiation using different enhancers and inhibitors added in a dose-responsive manner. Additionally, we showed how the optical phase shift could be used to quantify other morphological changes, including cell confluence and cell death. Taken together, these results point to DHM as being a rapid, robust, marker-free, and nonperturbing method, suitable for medium- to high-throughput screenings of adipocytic differentiation.

## MATERIALS AND METHODS

### Cell culture and adipocytic differentiation

OP9 cells (provided by T. Nakano, Kyoto University, Japan) were maintained in Minimum Essential Media α with GlutaMax^TM^ (Gibco, catalog no. 32561), 10% FBS (Gibco, catalog no. 10101), and penicillin/streptomycin (Gibco, catalog no. 15140) at 37°C and 5% CO_2_. The cells were split when subconfluent every 3–4 days. For every experiment, the cells were plated at confluence, i.e., 20.000 cells per cm^2^ (unless otherwise specified). Microscopy experiments were performed in 384-well plates, for which we plated 2,000 cells per well in a volume of 30 µl using an automatic liquid dispenser. Tested compounds were plated before the cells using the ECHO500 acoustic dispenser (Labcyte). Adipocytic differentiation was performed during 6 days using a differentiation cocktail containing dexamethasone (Sigma, catalog no. D4902; 10 µM), insulin (Sigma, catalog no. I0516; 5 μg/ml), and isobutyl-methylxanthine (I5879, 0.5 mM). The differentiation cocktail was added at double concentration in 30 µl of medium immediately after the cells were plated, making a final volume of 60 µl per well. For toxicity assessment, cells were incubated with gambogic acid, colchicine, taxol, and staurosporine in concentrations ranging from 1 nM to 30 μM.

### Microscopy

**Fig. 1.**
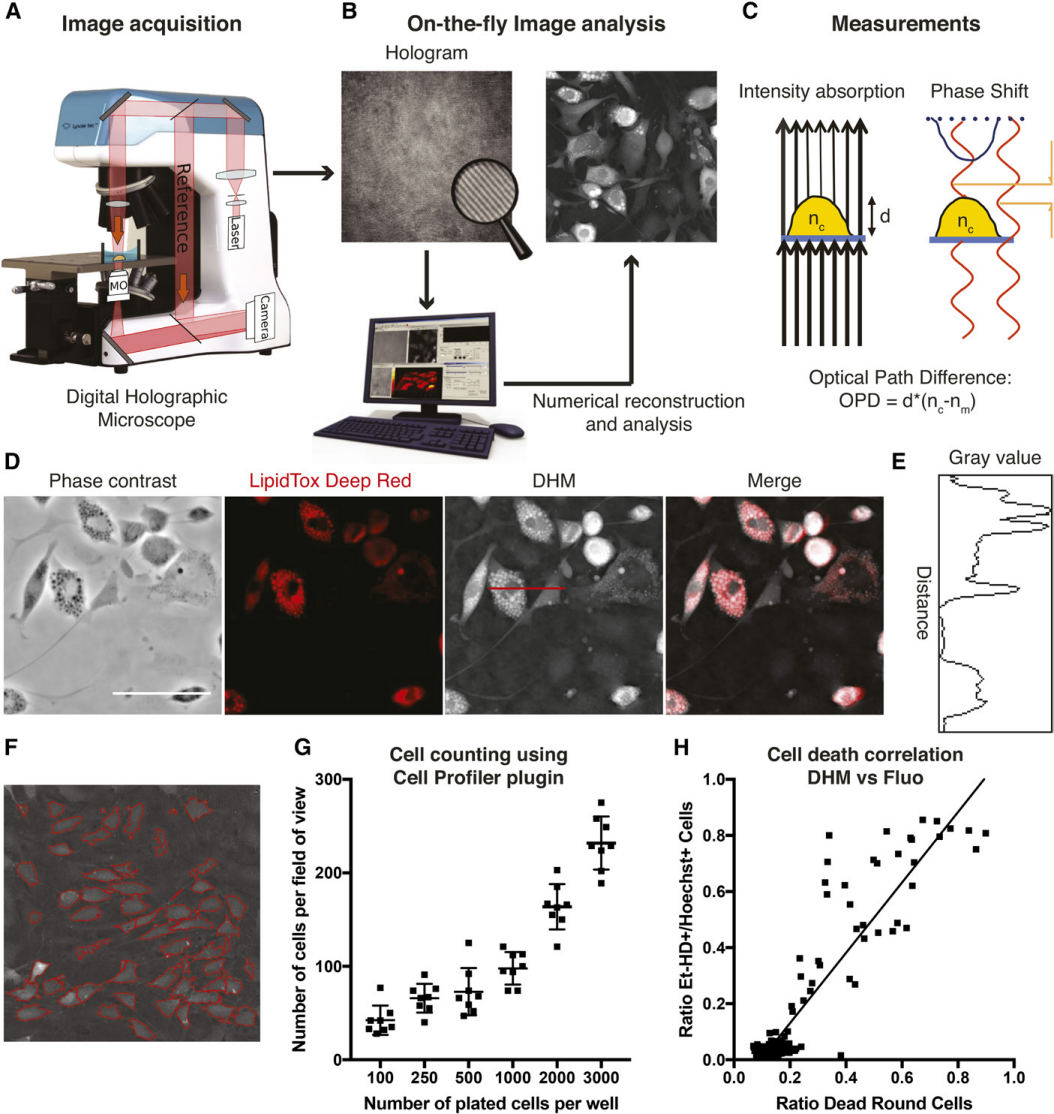
DHM is noninvasive and can be used to assess different features in lipid droplet accumulating cells. A: Schematic representation of DHM image acquisition. An infrared laser beam passes through the sample, while a reference beam passes in parallel without crossing the sample. B: Image reconstruction and analysis. The superimposition of both waves creates a hologram containing the phase shift information, which can then be mathematically reconstructed into a single-plane image. C: Information contained within the hologram. In addition to the phase shift information, DHM records the intensity absorption D: Superimposition of fluorescence lipid staining with DHM image. On the left are a digital phase contrast image (nonquantitative) and LipidTox Deep Red fluorescence images. On the right is the superimposition of LipidTox Deep Red signal with DHM signal. E: Signal intensity histogram of the cross-section seen as a horizontal red line passing through a lipid droplet-accumulating cell on the DHM image in D. F: CellProfiler software image analysis detects single cells, represented by their red outlines. Cells touching the borders are excluded. G: Cell counting using the aforementioned CellProfiler plugin. Increasing numbers of cells were seeded into a 384-well plate and counted from DHM images acquired 24 h after plating. H: Correlation of detection of OP9 dead cells exposed to various toxic compounds from DHM images as compared with fluorescence (Fluo) images from Et-HD and Hoechst 33342 stains. Dead cells were defined in the CellProfiler plugin as small and rounded with a high phase shift signal. Error bars are SD. Scale bar = 100 μm.

DHM technology and image reconstruction are described in detail elsewhere (15, 18, 23). DHM is a label-free interferometric microscopy technique, which provides a quantitative measurement of the optical path difference (OPD; related to the optical density of the cell). It is a two-step process where a hologram consisting of a 2D interference pattern is first recorded on a digital camera (**Fig. 1A**), and the quantitative phase images are then numerically reconstructed using a specific algorithm (Fig. 1B). The phase contrast in DHM images is quantitatively related to the OPD (Fig. 1C), expressed in terms of physical properties as:

(*Eq. 1*) $$OPD\left(x,y\right)=d\left(x,y\right)\times \left[n_{c}\left(x,y\right)–n_{m}\right],$$

where *d*(*x*,*y*) is the cell thickness, *n*_c_(*x*,*y*) is the mean z-integrated intracellular refractive index at the (*x*,*y*) position, and *n*_m_ is the refractive index of the surrounding culture medium. Simply, equation 1 means that the OPD signal is proportional to both the cell thickness (providing information about cell morphology) and the intracellular refractive index (providing information about the intracellular content of the cells, mostly protein and lipid content).

DHM systems generally use a low-intensity laser as light source for specimen illumination. Here, the 684 nm laser source delivers roughly 200 μW/cm^2^ at the specimen plane, approximately six orders of magnitude less than intensities typically associated with confocal fluorescence microscopy. With that amount of light, the exposure time is only 400 μs. An extensive quality control of DHM can be found in Rappaz et al. (24).

Digital holographic imaging was performed in black wall 384-well imaging plates (Corning, catalog no. 353962) using Transmission DHM® T1000 (Lyncée Tec, Switzerland). Plates were precoated with poly-ornithine (Sigma, catalog no. P3655; 100 mg/l) to prevent cell detachment. The cells were imaged live and without prior liquid manipulation using both a 10×/0.3 numerical aperture (NA) and 20×/0.4 NA microscope objectives. By taking four images per well, we covered almost one-fifth of the well surface (using the 10× objective). The best-focus phase images were reconstructed automatically in MATLAB (MathWorks) from the acquired holograms, and the average quantitative phase signal or OPD (16) was automatically measured using a fixed threshold value.

Fluorescence Images were taken with the InCell Analyzer 2200 (GE Healthcare) Imaging system. The neutral lipid droplets were stained with either LipidTox Deep Red (Life Technologies, catalog no. 34477) at 1:500 or Nile Red (Sigma, catalog no. N3013) at 10 μM. The nuclei were stained with Hoechst 33342 (1 µg/ml; ThermoFisher). For LipidTox imaging, the dyes were directly added to the wells, whereas for Nile Red imaging, the dyes were added with new medium. The wells were washed once with medium after 30 min, as recommended by the manufacturer. For toxicity assessment, dead cells were stained using ethidium-homodimer (Et-HD; Invitrogen, catalog no. E1169), and all cells were stained with Hoechst (catalog no. 33342, ThermoFisher), by directly adding the dyes into each well and imaging without any washing steps after 30 min incubation. As was done with DHM, four pictures were taken per well. Digital phase contrast images were additionally taken with the InCell Analyzer 2200.

### Image processing

For digital holographic imaging, the cells were first automatically segmented using a fixed threshold at a value slightly higher than the background to remove the contribution of noisy pixels. The average OPD was then measured within the segmented cells. Subsequently, the value of the four images taken per well was averaged and used as the quantified value for adipocytic differentiation. To count cell numbers or to quantify dead cells with DHM images, a segmentation tool for CellProfiler software (25) was used (Ridler Calvard’s algorithm). Cells were categorized to either “undifferentiated,” “adipocytic,” “dead,” or “errors” within the machine learning-based CellProfiler Classifier. The cells were subsequently counted automatically, using either the Random Forest or k-Neighbors classification algorithms, with cells at the borders of the images excluded from the analysis.

Fluorescence images were processed using the CellProfiler image analysis software. Uneven illumination gradients were corrected using the CorrectIllumination modules through a Gaussian blur. For LipidTox Deep Red signal quantification, the intensity was measured in a radius of 20 μm around each Hoechst-stained nucleus. For Nile Red signal quantification, a mask of the cytoplasm was first created using the faint membrane staining of Nile Red. The Nile Red fluorescence intensity of the lipid droplets was then measured within this mask, and the median of all cells was calculated for each image. For both Nile Red and LipidTox stains, the median fluorescence intensity was quantified within the masks, and the four pictures taken per well were then averaged. For Et-HD signal quantification, an automatic threshold was used to segment the cells, and a fixed threshold was used to set the separation between positive and negative cells. The ratios of Et-HD positive cells within all four images were calculated and averaged for each well.

### FACS

OP9 cells were plated in precoated 6-well plates at a density of 20.000 cells per cm^2^ and induced to adipocytic differentiation for 6 days. Prior to flow cytometric sorting, cells were stained with LipidTox Deep Red and propidium iodide (catalog no. 81845, Sigma-Aldrich) for 30 min, washed with PBS, and subsequently detached by incubating for 5 min in 0.05% trypsin-EDTA (Gibco, catalog no. 25300) at 37°C. The OP9 cells were then gently resuspended in PBS and sorted using the FACSAria Fusion Cell Sorter (BD Biosciences). They were collected into Eppendorf tubes, filtered using a 150 µm mesh, and subsequently plated into black wall 384-well plates at a density of around 4,000 cells per well. The settings for the side scatters (SSCs) and the forward scatters were based on the signal of noninduced OP9 cells as previously described (10).

### RT-PCR

OP9 cells were plated in precoated 24-well plates at a density of 20.000 cells per cm^2^ and induced toward adipocytic differentiation for 6 days as described above. RNA was extracted using ZR RNA Microprep (catalog no. R1060, Zymo) following the manufacturer’s instructions. A DNase I digestion step was included. cDNA was synthesized using the SuperScript VILO cDNA synthesis kit (catalog no. 11754-050, Life Technologies) following the manufacturer’s instructions. Quantitative real-time PCR was performed on QuantStudio 6 (Life Technologies) using the following primer pairs: *pparg*: forward (Fw): 5′-ACCACTCGCATTCCTTTGAC; reverse (Rv): 5′-TGGGTCAGCTCTTGTGAATG; *c/ebpa*: Fw: 5′-GTTAGCCATGTGGTAGGAGACA′ Rv: 5′-CCCAGCCGTTAGTGAAGAGT; *glut4*: Fw: 5′-GGCTTCTTCATCTTCACCTTC; Rv: 5′-TGGGTTTCACCTCCTGCTCT; *adipoq*: Fw: 5′-TGTTCCTCTTAATCCTGCCCA; Rv: 5′-CCAACCTGCACAAGTTCCCTT; *actb*: Fw: 5′-CTAAGGCCAACCGTGAAAAGAT; Rv: 5′-CACAGCCTGGA­TGGCTACGT; and *hprt1*: Fw: 5′-GCAGTACAGCCCCAAAATGG; Rv: 5′-AACAAAGTCTGGCCTGTATCCAA.

The geometric mean of both housekeeping genes (*actb* and *hprt1*) was used as reference to calculate the fold expression of each gene relative to the samples of noninduced OP9 cells 1 day after plating.

### Triglyceride content

OP9 cells were plated in precoated 24-well plates at a density of 20.000 cells per cm^2^ and induced to adipocytic differentiation for 6 days. The cells were washed once with PBS and homogenized in 300 μl of 10% Triton X (catalog no. M143-1L, VWR). Triglycerides (TGs) were solubilized by heating to 80°C in a water bath, cooling down to room temperature, and repeating, before storing at −80°C. After thawing, 25 μl of each sample was taken for the biochemical assay using Triglyceride Quantification Colorimetric/Fluorometric Kit (catalog no. K622-100, BioVision) following the manufacturer’s instructions in a 96-well black-walled plate. After 1 h reaction, the plate was read on an Infinite F5000 Spectrophotometer (Tecan, Switzerland) using the excitation/emission = 535/590 nm filter set for the fluorometric assay. The background signal was subtracted from all samples, and the TG concentration was estimated using the linear standard curve, which was not forced to intercept the *y* axis at *y* = 0.

### Protein content

The protein content was quantified in parallel using the same samples that were solubilized in 10% Triton X for the TG content measurements. Twenty-five μl of each sample was taken and measured using the Pierce BSA Protein Assay Kit (catalog no. 23227, Thermo Scientific) following the manufacturer’s instructions. After 30 min reaction, the absorbance was read on the spectrophotometer using the 560 nm filter. The protein concentration was estimated using the linear standard curve, which was not forced to intercept the *y* axis at *y* = 0.

## RESULTS

### DHM imaging is noninvasive and can be used to assess different features in lipid droplet accumulating cells

In this study, we show how the ability of DHM to detect morphology and refractive index in a nonperturbing manner makes this technique ideal to quantify lipid droplets in differentiating adipocytes.

We used the murine bone marrow-derived OP9 stromal cells (19). OP9 cells have been characterized as a useful model to study adipocytic differentiation thanks to their rapid rate of lipid droplet accumulation (20), even at high confluency or over long culture periods. When observing the signal intensity histogram of the cross-section of differentiating OP9 cells, both the cytoplasmic signal and the highly refractive lipid droplets are clearly distinguishable from the background noise (Fig. 1E). This signal can also be used to assess different features of the cells including cell size, morphology, and confluence. Using a machine learning-based CellProfiler Classifier, we were able to categorize cells and assign them to “undifferentiated,” “adipocytic,” or “dead” phenotypes. Undifferentiated cells were defined as large and with low OPD signal, adipocytic as large with high OPD signal, and dead cells as small and round with a high OPD signal. We plated OP9 cells at different concentrations, imaged them after 24 h, and counted individual cells (result of the CellProfiler segmentation in Fig. 1F). As expected, a higher proportion of cells were detected in wells, which were initially seeded at a higher concentration (Fig. 1G). The exponentiality of the curve may represent the proliferation kinetics of cells; a higher initial cell number will predict a greater increase in cell numbers after 24 h. To assess the capacity of our workflow to detect dead cells, we first incubated OP9 cells with different known toxic compounds and acquired DHM images. The cells were then stained with Et-HD and Hoechst 33342 (for dead cells and total cells, respectively) and imaged using the InCell Analyzer 2000 (GE Healthcare). We plotted each well and compared the fraction of Et-HD-positive cells from the fluorescence images with the fraction of dead rounded cells detected in DHM images. This correlation is linear with *R*^2^ = 0.85, *P* < 0.0001 (Fig. 1H). The variability is increased with a higher proportion of dead cells, which may be due to dead cells detaching from the bottom, characterized by a shift toward the right of the correlation curve.

### DHM can be used to assess adipocytic differentiation

**Fig. 2.**
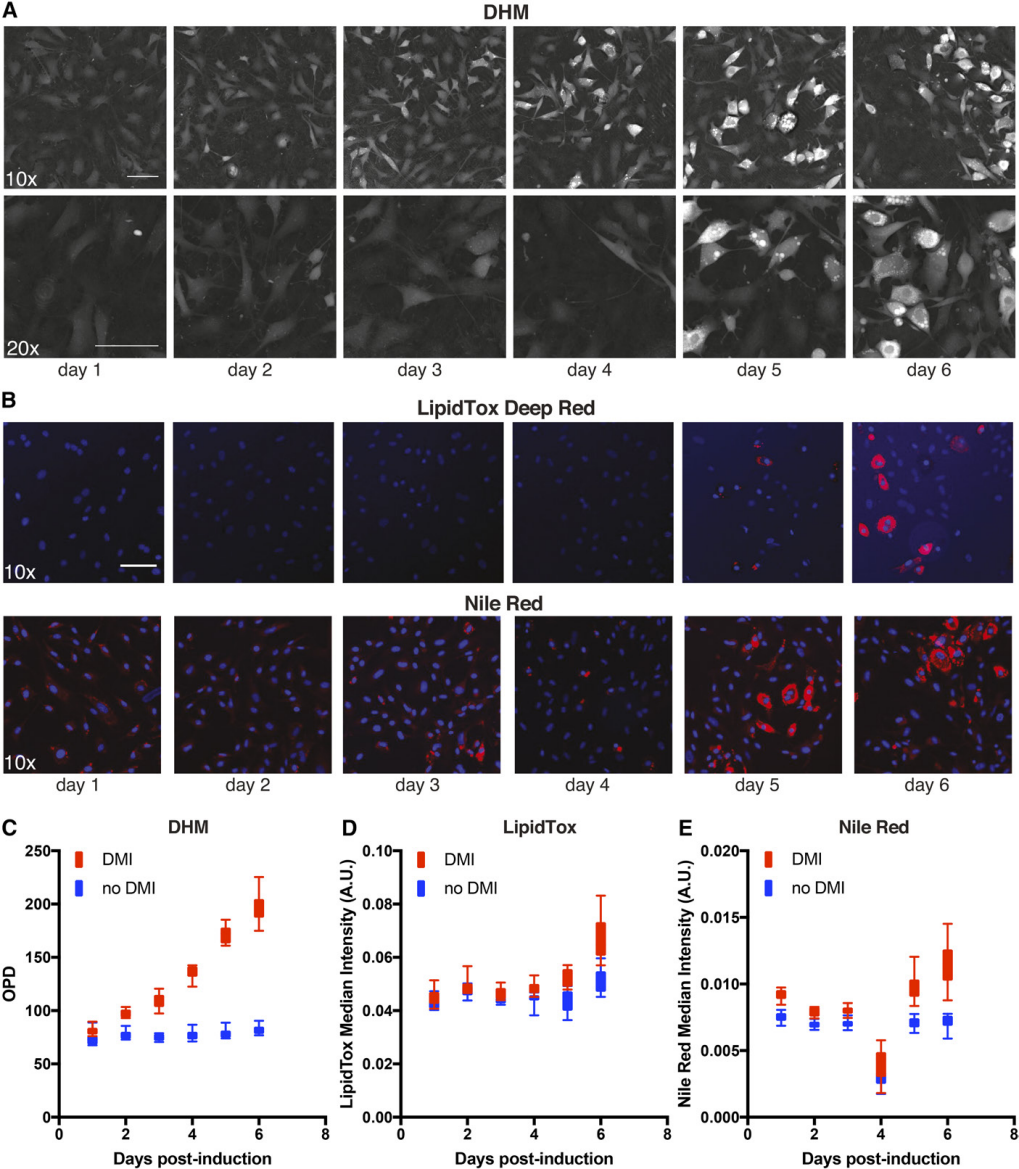
Six-day time lapse of OP9 cells induced to differentiate toward adipocytes: Comparison of DHM and fluorescence microscopy. A: DHM images from day 1 to 6 postplating and induction. Top, 10× bottom, 20×. B: Fluorescence images taken from day 1 to 6 postplating and induction. Blue is Hoechst 33342, and red is either LipidTox Deep Red (top) or Nile Red (bottom). C: OPD values of OP9 cells induced to differentiate (red) versus noninduced OP9 cells (blue). D, E: LipidTox Deep Red and Nile Red median signal intensities in both induced and noninduced OP9 cells. Note the error during the Nile Red staining procedure on day 4 postinduction of adipocytic differentiation. *R*^2^ = 0.6 for OPD *vs.* LipidTox Deep Red and *R*^2^ = 0.54 for OPD *vs.* Nile Red. Error bars are SD. Scale bars = 100 μm.

To assess the capacity of DHM imaging to recapitulate the quantification of lipid droplets, we compared it to classic imaging of stained neutral lipids. Specifically, we compared the OPD signal with the fluorescence signal of both LipidTox Deep Red and Nile Red along a 6 day adipocytic differentiation period (**Fig. 2**). Due to the preadipocytic nature of OP9 cells, around 5% of the cells spontaneously differentiated toward adipocytes when left confluent over 6 days (data not shown). The DHM images detected changes in differentiation after 2 days in culture (Z′ = −2.2, 0.06, 0.23, 0.53, 0.65, and 0.6 for days 1–6, respectively; Fig. 2A, C), while it took at least 5 days to see any detectably stained lipid droplets by fluorescence images (Fig. 2B, D, E). Unfortunately, on the day 4 datapoint, the Nile Red staining did not yield sufficient cytoplasmic membrane staining, which, in turn, revealed a low overall intensity of lipid droplet quantification and thus revealed an outlier datapoint (Fig. 2E). This common artifact highlights the advantages of an alternative nonperturbing method to quantify lipid droplet accumulation. DHM reduces the number of variables introduced into each experiment, thus increasing the robustness and reproducibility of lipid droplet quantification. The DHM-derived OPD signal correlated nonlinearly with both the LipidTox Deep Red and Nile Red Fluorescence signal with *R*^2^ = 0.6 and *R*^2^ = 0.54 respectively, *P* < 0.0001 (data not shown).

**Fig. 3.**
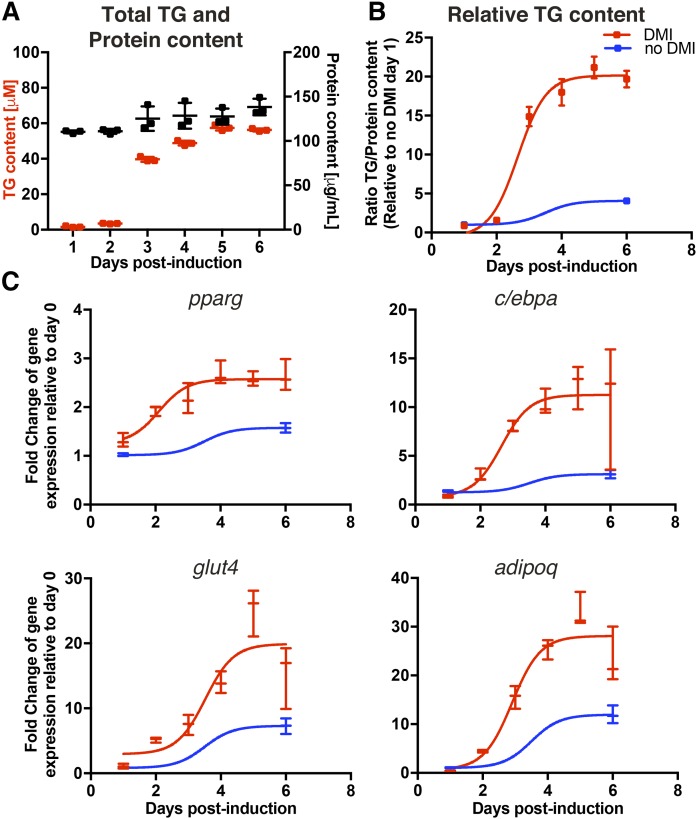
A: Total TG and protein content of OP9 cells during the 6 day adipocytic differentiation. Quantification is based on the standard curve from the manufacturer’s control. B: Relative TG content over protein content during the 6 day differentiation. C: The transcriptional profile of relevant genes involved in the adipocytic differentiation of OP9 cells. Values are normalized to noninduced OP9 cells 1 day postplating and the geometric mean of two housekeeping genes: *hprt1* and *actb*. Error bars are SD.

In parallel, we quantified biochemically both the TG and the protein content over the course of the 6 day adipocytic differentiation (**Fig. 3A**, B). As expected, the protein content stayed relatively constant, while the TG content increased almost 30-fold, reaching the plateau on day 4–5 postinduction. For comparison, the TG content of OP9 cells increased almost 5-fold over 6 days without induction of differentiation. We also analyzed the transcriptional profile using a set of known markers of adipocyte differentiation and function. We quantified two transcription factors (*pparg* and *c/ebpa*) and two functional adipocyte markers (*adipoq* and *glut4*). Owing to the preadipocytic nature of OP9 cells, all gene transcripts were upregulated after 6 days, even without induction of differentiation (Fig. 3C). Induction of adipocytic differentiation significantly increased the transcription of all analyzed genes (*pparg* increased 2.5-fold; *c/ebpa* increased ∼10-fold; *glut4* increased ∼20-fold; and *adipoq* increased ∼30-fold). All gene transcripts measured plateaued at 5 days of differentiation.

**Fig. 4.**
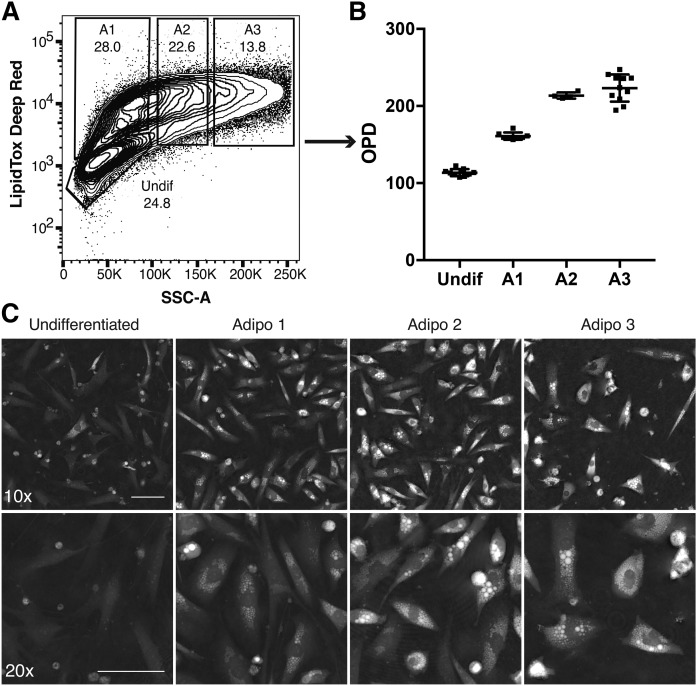
OPD signal of FACS-sorted OP9 cells induced to differentiate toward adipocytes during a time period of 6 days. A: Flow cytometric gates were chosen along the SSC signal (representing different stages of lipid accumulation), sorted separately, and subsequently plated into a 384-well plate. B: Average OPD signal of sorted OP9 cells 24 h after plating. Images without cells were removed from analysis. C: Representative DHM images of sorted OP9 cells. Top, 10× bottom, 20×. Error bars are SD. Scale bars = 100 μm.

Furthermore, we stained OP9 cells with LipidTox Deep Red and sorted them based on their granularity as previously shown (10, 22) (**Fig. 4A**). Note that the dynamic range and resolution of cells exhibiting different levels of adipocytic differentiation was higher in the SSC in linearity than along the fluorescence axis in exponentiality. The cells were plated and underwent DHM imaging 24 h later (Fig. 4C). As expected, cells with a higher initial SSC signal also had a higher OPD signal. Of note, cells sorted from the A3 gate (representing the most mature adipocytes) exhibited less adherent behavior, explaining why the cell density was lower on their corresponding images. These mature A3 cells also appeared rounder, although their OPD signal was not significantly different (Fig. 4B).

### DHM imaging is robust and high-throughput

**Fig. 5.**
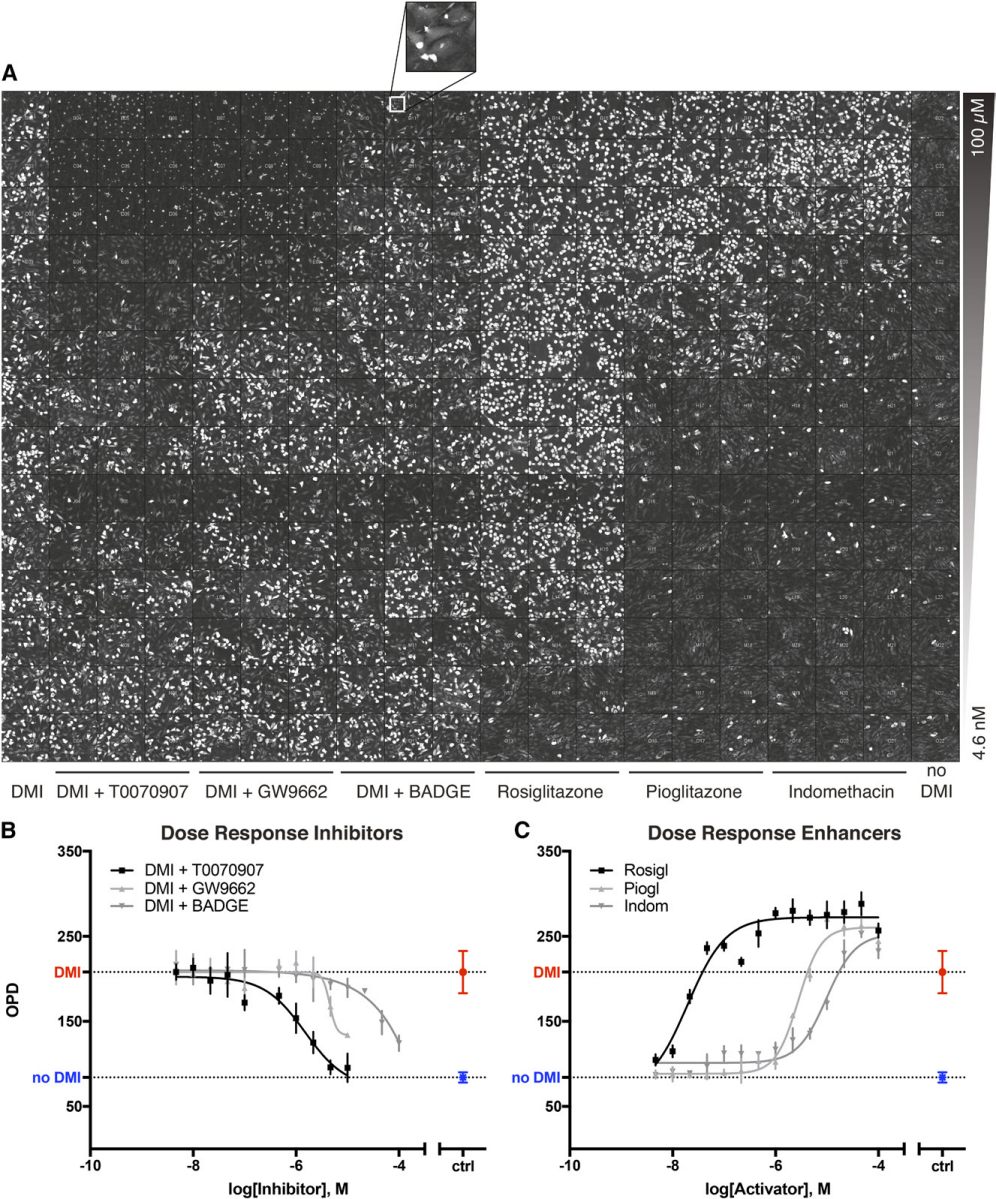
DHM imaging can be used to identify inhibitors and enhancers of adipogenesis in a high-throughput manner. A: Snapshot of a 384-well plate presenting a montage of DHM images of OP9 cells incubated with different PPARγ antagonists and agonists at different concentrations (one image per well). On the left half of the plate, OP9 cells were induced to differentiate to adipocytes while incubated with different PPARγ inhibitors (T0070907, GW9662, and BADGE). On the right half of the plate, OP9 cells were not induced with the standard differentiation cocktail, but incubated with different PPARγ agonists (rosiglitazone, pioglitazone, and indomethacine). The small inset on the top shows dead cells B: Dose-response curves of different tested PPARγ inhibitors. C: Dose-response curves of the different tested PPARγ agonists. Dashed lines represent baseline OPD values for induced (DMI) or noninduced (no DMI) OP9 cells. Error bars are SD.

Finally, we assessed the capacity of DHM imaging to identify modulators of adipocytic differentiation in a high-throughput manner. We cultured OP9 cells with known enhancers (rosiglitazone, pioglitazone, and indomethacin) of adipocytic differentiation (**Fig. 5A**, right) as well as with known PPARγ inhibitors (T0070907, GW9662, and BADGE; Fig. 5A, left) in a dose-responsive manner in a 384-well plate. Inhibitors of adipocytic differentiation were plated in combination with the adipogenesis induction cocktail DMI to test the strength of their inhibitory capacities. The compounds were tested in 14 different concentrations ranging from 100 µM (Fig. 5A, top) to 4.6 nM (Fig. 5A, bottom). As visualized from the images, PPARγ inhibitors increasingly prevented adipocyte differentiation at higher concentrations, while adipogenesis enhancers triggered adipocytic differentiation more efficiently at higher concentrations. As shown in the high-magnification insert, PPARγ inhibitors are toxic at high concentrations (>10 µM), making the cells appear round with a high phase shift signal (Fig. 5A). T0070907 presented the most potent effect of the three tested inhibitors and could revert the OPD signal back to control levels before reaching toxic concentrations. Specifically, T0070907 exhibited an IC_50_ = 1.49 µM; GW9662, IC_50_ = 4.49 µM; and BADGE, IC_50_ > 100 µM (Fig. 5B). Within the enhancers of adipocyte differentiation, rosiglitazone exhibited the most potent effect with an IC_50_ = 19.5 nM, while pioglitazone and indomethacine had IC_50_ values of 1.23 and 2.77 µM, respectively (Fig. 5C).

## DISCUSSION

OP9 cells are bone marrow-derived MSCs/preadipocytes that can efficiently differentiate into adipocytes (19, 20, 26). Their differentiation process can be studied from day 2 and is characterized by the early expression of PPARγ, C/EBPα, C/EBPβ, and SREBP1 (20) transcription factors. PPARγ is a master regulator of adipocytic differentiation. It translocates to the nucleus upon activation and drives adipocytic commitment, while preventing the cells from differentiating along the osteogenic lineage by directly inhibiting *runx2* transcription (27). In addition, OP9 cells are nonclonal, allowing us to evaluate heterogeneity within differentiating cells.

Label-free and noninvasive imaging of in vitro differentiating adipocytes is crucial to limit the adverse effects caused by staining, washing, and other liquid manipulations used in fluorescence microscopy. In this study, we have shown that the quantitative phase images generated by holographic microscopy are able to provide a variety of information on cell morphology, including cell confluence, cell death, and lipid droplets, in a nonperturbing manner, thus reflecting true physiologically relevant measurements. Using the freely available machine learning plugin for CellProfiler, we were able to categorize OP9 cells into “adipocytic,” “undifferentiated,” or “dead” phenotypes. The information given on cell confluence and categorization can also be envisioned to study hypertrophy versus hyperplasia behavior in differentiating adipocytes. To quantify adipocytic differentiation in bulk, simply the OPD within the cytoplasmic areas provided an accurate quantification of lipid droplets. We correlated the OPD measurement to fluorescence microscopy: Et-HD/Hoechst 33342 costaining for cell death and LipidTox Deep Red/Hoechst 33342 costaining for lipid droplets. Variations caused by liquid manipulations, illumination irregularities, dependence on dye concentrations, staining times, and bleaching are key issues in fluorescence microscopy and therefore strongly compromise quantification accuracy and robustness. DHM proved to be more sensitive than fluorescence microscopy, allowing detection of differences in adipocyte differentiation up to 3 days earlier. Another key feature of DHM is that multiple independent time points can be measured; the same cells were imaged in a time lapse without any of the compromises of fluorescence microscopy. In our study, we took four pictures per well on a 384-well plate, which took roughly 10 min. The ability to digitally refocus on the cells postimaging speeds up the process of data collection and adds to the simplicity of the imaging procedure toward a higher throughput. We also compared DHM imaging with flow cytometry, another validated strategy of adipogenesis quantification. In this case, DHM quantification also correlates with the flow cytometric measurement. However, within late-stage differentiating adipocytes, the increase in OPD signal does not seem to increase significantly, which may be due to floating cells not being measured at late differentiation stages with DHM. Quantification of lipid droplets using flow cytometry is also limited by variabilities in cell collection, cell floating, and the fragility of terminally differentiated adipocytes. We further tested the robustness of DHM to assess lipid droplet accumulation in differentiating adipocytes by incubating OP9 cells with PPARγ agonists and antagonists in a dose-responsive manner. We were able to calculate the IC_50_ for each of the tested compounds and showed that, with high doses of PPARγ agonists, adipocytic differentiation can be pushed further than with our standard differentiation cocktail. Taken together, our study shows that this novel, nonperturbing imaging method can be used to assess a variety of morphological features without the need of developing lengthy and tedious staining protocols. The DHM, besides reducing costs in consumables, is therefore a powerful tool envisioned for future drug screenings and mechanistic studies on adipocyte differentiation.
