# Corrigendum: High-throughput, nonperturbing quantification of lipid droplets with digital holographic microscopy

**DOI:** 10.1016/j.jlr.2022.100291

**Published:** 2022-10-03

**Authors:** Vasco Campos, Benjamin Rappaz, Fabien Kuttler, Gerardo Turcatti, Olaia Naveiras

59 (2018) PAGES 1301–1310

In the original article, there was an error in the Material and Methods, p.1302, section “Cell culture and adipocytic differentiation“. The dexamethasone concentration was stated 10 μM while it should be 1 μM. The correct sentence should read as follows: “Adipocytic differentiation was performed during 6 days using a differentiation cocktail containing dexamethasone (Sigma, catalog no. D4902; 1 μM), insulin (Sigma, catalog no. I0516; 5 μg/ml), and isobutylmethylxanthine (I5879, 0.5 mM).”

The authors state that the correction does not affect the results and conclusions of the paper.

